# Report of Surgical Cases in the Buffalo General Hospital

**Published:** 1863-04

**Authors:** Julius F. Miner


BUFFALO

Medical and Surgical Journal

VOL. II. APRIL, 1863. NO. 9.

### ORIGINAL COMMUNICATIONS.

### ART. I.—*Report of Surgical Cases in the Buffalo General Hospital.* By Julius F. Miner, M. D.

*Abscess.*—Under this head are placed only those cases which are regarded usually as phlegmonous abscess, since the specific, scrofulous, or other forms, are only considered as part of the general disease with which they occur. The only point to which we desire to call attention in connection with abscesses, is their proper treatment, not desiring to speak of the usual classes into which they are divided, or consider any of the various causes by which they are produced, or diseases with which they are often connected.

It has been regarded by many surgeons, from Abernethy until the present time, that opening of abscess should be so made as to exclude atmospheric air, or at least much has been said and written upon the necessity of this in some cases, psoas, hepatic, scrofulous and some other forms of the disease. When connected with or arising from disease of some of the larger joints, it has been regarded as especially important, if the pus is allowed to escape that air is not allowed to come in contact with the diseased surfaces by which it has been produced. The view has been maintained, that air if admitted to the pyogenic membrane would cause severe constitutional disturbance, or if in contact with purulent matter has a tendency to produce decomposition, and thus the severest consequences. It is not intended to consider at all the various theories which have been proposed, or the arguments by which they have been sustained, but simply to call attention to the subject for the purpose mainly of saying, that so far as yet observed there is really no good reason for believing that atmospheric air when admitted to the lining membrane of an abscess is productive of any injury whatever. Pus, wherever detected may be, indeed should be, allowed free exit; and there is no danger in making opening upon it, the danger is in not allowing its early escape. Pus, when detected during the progress of scrofulous disease of the hip joint, vertebræ, knee, or other structure, should be discharged, and its removal will be attended by relief more or less well marked, in all instances. If there is no danger in these cases, there can hardly be supposed to be any objection to making free opening for the escape of pus under any circumstances, or necessity of attempting to so make it, as to prevent the ingress of air, which if attempted would in almost all cases. sooner or later, result in complete failure. It is sometimes recommended to leave this operation for nature to perform, thus allowing her to accommodate herself to the impressions which are to be made upon her. This is generally error, but it is in the right direction, for it shows greater trust in nature's workings, and less confidence in the benefits of operative interference on the part of the surgeon. A rule, however, admitting of very few, if indeed of any exceptions, may be briefly stated; pus, wherever detected, should be immediately allowed free exit.

*Gun-shot Wounds.*—The two cases presented were both of long standing. One was a soldier wounded some six months previous to admittance by a ball passing through the muscles of the lower part of the thigh, in very near proximity to the femoral artery and sciatic nerve, producing sufficient impression upon the latter to partially paralyze the leg and foot He had also received injury of the foot by shell, and the first and second toes had been amputated in consequence. The circulation in the foot was exceedingly feeble, and ulcerations were common in the cicatrix, while at one time the third toe turned very dark, and for several days seemed likely to separate and fall from its attachments. Elevating the foot, and application of yeast poultices constituted the treatment, and recovery took place with improved condition of system, though the paralysis remained unchanged.

The second case was that of a soldier wounded in the last battle of Bull Run. This was the private patient of Dr. Storck, and still continued under his care. The wound had been received in the left side, the ball striking the sixth rib and producing necrosis of the middle portion of that bone.

The suppuration was very profuse, the emaciation and debility extreme; probe detected loose and denuded bone; several small fragments had already been discharged. By request of Dr. Storck I assisted in the removal of the diseased portion, with the hope that suppuration would be lessened, and the efforts of nature which had been vigorously instituted would be assisted the earlier to rid itself of the irritation and exhaustion thereby ocsasioned. The case still remains under treatment, and the probable termination is hardly yet determined. So far as the effect of the removal of the dead bone is concerned, it may be fairly said to have been decidedly beneficial, though the discharge for a few weeks was rather increased, and perhaps the exhaustion augmented. This case has many important features, and would be described more in detail had it been under care as a hospital patient, strictly speaking. It has been referred to thus briefly since it enters the record, and could not be wholly omitted.

*Fractures.*—Two cases only are of importance, the others being simple fracture without unusual features of any kind.

The first of these was fracture of the tibia and fibula about the middle, in a healthy young man, 28 years old, who had never had disease of any kind. He was admitted to the marine ward, under the care of my predecessor, Dr. Eastman, about the first of September, 1862. The leg was dressed in the usual manner with bandage and side splints, which retained the bones in proper position very satisfactorily. There was not much pain or inflammation, and everything seemed to progress in favorable manner, not exciting suspicion of any trouble whatever. Six weeks after the accident when the dressings were removed for examination and re-adjustment it was observed that there was no union, the bones moved freely upon each other, and the leg was nearly as flexible as when first fractured. The leg was now dressed in immovable starch bandage, supported by pasteboard splints, and allowed to remain without motion for six weeks more, at which time the dressings were again removed. Motion was the same, and there was no indications of bony union taking place; the ends could be freely moved at the seat of fracture, and the leg bent iu all directions.—The general health was good, confinement not having impaired his robust constitution. After much consideration and consultation with my colleagues of the active staff, about the fourteenth week after the fracture, the ends of the bones and the ligamentous structure between their ends were bored with a drill, in as many directions as possible without making puncture through the skin and integuments at but a single point. The drill was made to pass nearly through the bone, or the distance of an inch in depth, and the drill was about the sixteenth of an inch in diameter.—This manner of operating for ununited fracture was first adopted and advocated by Prof. Brainard of Chicago.

The leg was placed in the starch splint in which it had rested before, which had been made a perfectly fitting case, and opened upon the upper part, allowing its removal when necessary. Inflammatory action now commenced with considerable constitutional sympathy. Pulse increased to 120 per minute; tongue became dry with thirst; chill, heat, and perspiration alternating with each other. Pain was at times quite severe, making anodynes necessary to relieve it, and procure sleep The disturbance was so great as to convince us that the operation of boring between ununited bones and into their structure, was not so simple and pleasant as we had imagined, and that greater risk attended it than would hardly be supposed until practiced. The object in making this attack upon the bone, was to produce a certain necessary amount of inflammatory action; in this case we produced a seemingly very unnecessary amount of inflammation. and yet there was very little suppuration or other unpleasant effect resulted from it, and it passed off very kindly and gradually. The point, which is however to be especially noticed, may be briefly stated in few words. In six weeks we had the great satisfaction of finding bony union; the operation was signally successful. Extensive deposit had taken place around the fractured ends of the bones, the sharp points being thus smoothed off or concealed, and when the small amount of suppuration had ceased, it was found to our great gratification, that complete bony union had taken place.

The conditions usually regarded as causes of non-union after fracture were in this case wholly absent. The patient was a robust, healthy, young man; had no hereditary or acquired constitutional disease, so far as could be ascertained. He was quiet and free from all disturbing influences.—The fracture was early adjusted and properly retained in place, and there were no causes operating to produce this result other than what were completely concealed from observation; possibly, however, were present and consisted either in separation of the ends of the bone by some muscular or fibrous tissue, or in constitutional causes which were concealed from view. From careful observation of several other cases within the last few months, as well as the one above described, it has appeared almost certain that the causes of non-union after fracture are not known, and that the usual influences supposed to produce such accident are very often absent, and if present in most unmistakable form, fail to produce such a result.—This opinion can be sustained by some remarkable facts recently under observation, where bones did not unite when every condition seemed most favorable; and again on the other hand where bony union did rapidly take place, while the most potent influences both of local displacement and motion, conjoined with constitutional dyscrasia of malignant and fatal type were in active and manifest operation.

*The second* case of fracture we propose to describe was compound comminuted fracture of tibia and fibula, about the middle portion of these bones, with great destruction of the soft parts. It was caused by being run over by the wheel of a wagon, heavily loaded with stone. The patient was a strong, healthy laborer, aged 35 years. The leg was temporarily dressed by Dr. T. T. Lockwood, and sent by him to the hospital for care.

Five days after admittance the ends of the bones were bare, denuded of periosteum and blackened; very much resembling bones after gun-shot injury. Some small pieces were loose in the wound, and were removed.—The tibia was broken in such manner that the fragments were constantly slipping out of contact with each other and being blackened and necrosed, exsection of the lower fragment was made, something like an inch of sharp point removed, thus leaving the bone healthier and more perfectly in contact with the upper fragment. The constitutional disturbance is not great, though at the time our last record was made, the case looked unfavorably since the periosteum was separating and death of bone seemed extending with profuse discharge from the slough. The hope of saving the leg is not yet wholly abandoned.

*Diseases of Bones and Joints.*—The only case worthy of any especial mention and not included in any other class, was that of a young woman twenty-five years old, who had strumous disease of the knee-joint and caries of the ramus of the ischium. It had been of four or five years' standing, and necrosed portions of bone had separated from the ischium and been discharged from the opening, which was situated near the labia, having large and direct communication with the diseased bone. The knee-joint was greatly enlarged, with fistulous openings communicating with the bone and with the structures around the joint. The probe passed down upon the periosteum, but did not detect denuded bone. There was constant discharge from the sinuous openings, but it was not very profuse. The leg was flexed to nearly a right angle with the thigh, but had been in this condition for only six or eight months previous to which she had been able to walk with more or less facility. The general health was good, considering how long time she had suffered, and there was no pulmonary complication. She had the appearance of comfortable health, and aside from the conditions we have described was very well, suffering but little pain from the knee, and not any from the ischium. There was some motion in the knee-joint, and the flexion of the leg upon the thigh was due to contraction of the ham-string muscles.

The motive for giving this full description is to speak briefly upon the practical question of treatment proper in this and similar cases. It does not appear from the examination that the articulating surfaces of the joint are diseased, yet it is not unreasonsble to suppose that more or less inflammation and disease are actually present, and the question of extension involves an estimation of any risk of increasing this inflammation. It seemed to be a very general opinion of the medical men who examined the knee, that there was great risk of increasing the disease by any forcible or gradual extension of the leg, and that if it was extended, little, if anything, would thereby be accomplished towards either relief or cure. It would not be suggested with any expectation of thereby removing any disease of the bone, but simply for the purpose of placing the joint in more natural position, possibly of allowing the better use of the leg, and thus of partially relieving the more important disabilities arising from the disease, trusting to nature for more perfect restoration. Amputation of such joints was formerly quite unhesitatingly advised, but at present it would hardly be considered; the condition does not justify it. More recently exsection would be thought the more feasible method, if indeed any operative interference was justifiable. The caries of the ischium, however, would perhaps prove sufficient objection to any operation for removal of the disease, and we are narrowed down to the general plan of tonic medication, and whatever may be done for the relief of the deformity and improvement in the use of the leg.

From want of fortitude and resolution on the part of the patient, no very successful effort was made to place the leg in natural and useful position; the opportunity for such effort is not yet lost and confidence in its safety and practicability is unabated.

*Varicose Veins.*—The operation practiced for radical cure of varicose veins consisted in injecting into them a dilute solution per sulphate of iron, while pressure was made both above and below the point of injection by ligature or the finger of an assistant. By this means instantaneous coagulation of the blood was produced, and consequent complete obstruction of the vein. The coagulum thus formed remains for several days, and is usually absorbed; sometimes it suppurates, and occasionally when remaining hard for some time, it is well to open down upon and remove it. The operation, if skillfully performed with proper instrument, is nearly painless; the obstruction is certain and instantaneous, and the risk of unpleasant consequences must be very small, though great experience is necessary before it can be pronounced absolutely free from risks. In comparison with the operation of ligating the veins, the chief advantages must consist in less pain, and greater safety. The first we certainly secure, while it appears probable that the much less disturbance of the coats of the vessels would be attended by greatly diminished liability to phlebitis, absorption of purulent secretion, or other accident which has sometimes followed the operation of ligating varicose veins.

The efficiency of this operation in relieving the condition for which it is practiced, is unsurpassed. One of the cases under treatment, included under this head, was very severe, the anterior portion of the leg was completely covered by the vascular tumor thus made, appearing more like an aneurism by anastomosis, than varicose veins. This condition of the vessels disappeared as by magic, and the results of the blocking up of the principal veins was truly remarkable. It is usually recommended to place the leg after operation in a large poultice of flax-seed or slippery elm, and continue this for a week or more. The patient should be placed in horizontal position for a few days, and when walking it is well to support, for a time, the vessels, by applying a bandage or wearing an elastic stocking.

(TO BE CONTINUED.)

